# Methotrexate Treatment in Children with Febrile Ulceronecrotic Mucha-Habermann Disease: Case Report and Literature Review

**DOI:** 10.1155/2015/357973

**Published:** 2015-08-27

**Authors:** Isil Bulur, Hilal Kaya Erdoğan, Zeynep Nurhan Saracoglu, Deniz Arık

**Affiliations:** ^1^Department of Dermatology and Venereology, Faculty of Medicine, Osmangazi University, Eskişehir, Turkey; ^2^Department of Pathology, Faculty of Medicine, Osmangazi University, Eskişehir, Turkey

## Abstract

Febrile Ulceronecrotic Mucha-Habermann disease is a rare and potentially fatal variant of pityriasis lichenoides et varioliformis acuta and is characterized by high fever, constitutional symptoms, and acute oncet of ulceronecrotic lesions. We present an 11-year-old male with Febrile Ulceronecrotic Mucha-Habermann disease who was cured with methotrexate and review the use of methotrexate for this disorder in the pediatric age group with the relevant literature.

## 1. Introduction

Pityriasis lichenoides et varioliformis acuta (PLEVA) is a rare idiopathic dermatosis. Febrile Ulceronecrotic Mucha-Habermann disease (FUMHD), first defined by Degos et al. in 1966, is a severe variant of PLEVA that is characterized by destructive ulceronecrotic lesions and frequently accompanied by systemic findings [1].

Systemic steroids, oral antibiotics (erythromycin, tetracycline), phototherapy, and immunosuppressive agents are used for treatment but the results are mostly in the form of case reports. Methotrexate treatment has been reported as an effective option in a few cases in the literature. We report here an 11-year-old boy with FUMHD who was treated with methotrexate.

## 2. Case Report

**Figure 1:**
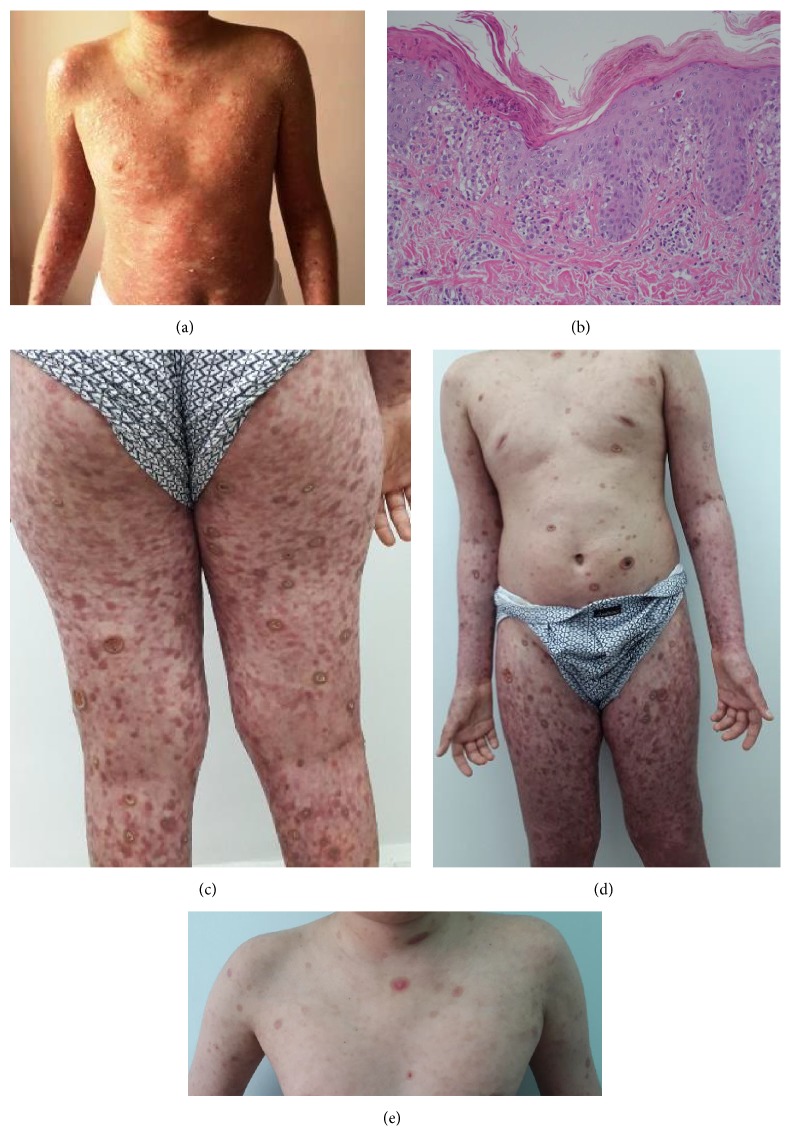
(a) Erythematous macules, papules, and pustules on the trunk. (b) Histopathology of the skin lesion showing parakeratosis, extension of infiltrate into epidermis, vacuolization of basal layer with necrotic keratinocytes, and mixed inflammatory cell infiltration around the vessels and in the upper dermis (HE ×200). (c) Necrotic ulcers, erythematous papules, and plaques on the limbs. (d) Clinical appearance after six doses of methotrexate. (e) Clinical appearance five months after the patient's initial visit.

An 11-year-old male presented at our clinic with a 15-day history of body rash. There was no history of medication use and drug or food allergies. He had a history of Guillain-Barre syndrome at the age of 9 years. Dermatological examination revealed a widespread polymorphic rash with erythematous macules, papules with a central punctum, and pustules on the trunk and extremities (Figure 1(a)). There was not any involvement in oral mucosa and conjunctiva. Laboratory tests revealed normal complete blood count, erythrocyte sedimentation rate, C reactive protein, and biochemistry values. Serologies for* hepatitis B virus, hepatitis C virus, HIV, cytomegalovirus (CMV), rubeola, toxoplasma, parvovirus, *and* herpes simplex virus* were negative.* Rubella, varicella, *and* Epstein–Barr virus* were negative for IgM but positive for IgG, suggesting a past infection. Serum IgA and IgM were within the normal range and IgG was slightly decreased (610 mg/dL, normal: 700–1600 mg/dL). Analysis of peripheral lymphocyte subsets revealed slightly decreased numbers of CD3+CD4 cells (21.2%, normal: 30–60%). The percentage of B cells (CD19+) was slightly elevated (35.8%; normal 1–35%).

The skin biopsy taken from the body lesion revealed parakeratosis in the epidermis with neutrophil groups within, mixed inflammatory cell infiltration around the vessels and the interstitial area in the upper dermis, and vacuolar degeneration in the basal layer (Figure 1(b)). We diagnosed our patient with PLEVA in the presence of these clinicopathological findings and started oral methylprednisolone 32 mg/day and oral erythromycin 500 mg 2 times a day in addition to topical supportive treatment. However, we noticed an exacerbation of the patient's lesions on the 10th day of treatment. The erythematous papular lesions covered almost all the body including the face and extremities, together with an AST value of 73 U/L (normal: 0–37) and ALT value of 262 U/L (normal: 0–41). We discontinued the erythromycin treatment and continued with methylprednisolone 48 mg/day and topical supportive treatment. The liver function test results recovered to normal limits 10 days after discontinuation of erythromycin, but the patient continued to develop new ulceronecrotic lesions (Figure 1(c)). The second biopsy taken from the new developing lesion was also consistent with PLEVA, and methotrexate 15 mg/week was added to the systemic steroid treatment. The systemic steroid treatment was gradually tapered and stopped. There was marked improvement in the lesions at the 6th week of methotrexate treatment (Figure 1(d)). Methotrexate was stopped after 5 months. We did not observe any secondary side effect due to the methotrexate treatment. The lesions healed with a postinflammatory hyperpigmentation and hypertrophic scar (Figure 1(e)).

## 3. Discussion

PLEVA is characterized by acute onset of scaly erythematous papules that might become vesicles and pustules with hemorrhagic necrosis and ulceration [2]. FUMHD is a rare and potential fatal form of PLEVA with a severe course and ulceronecrotic involvement [3]. PUBMED data reveal that only a limited number of pediatric FUMHD cases have been reported, and there is no clear treatment algorithm in this disorder.

Topical corticosteroids, immunomodulators, and oral antibiotics (erythromycin, tetracycline) have been used as a first-step treatment and PUVA and UVB as a second-step treatment for PLEVA, while systemic steroid, methotrexate, cyclosporine, dapsone, and acitretin are third-step treatment [2]. However, third-step treatment for PLEVA should be started as soon as possible in FUMHD cases [3].

When we reviewed the literature data, we observed that methotrexate treatment has been used in 15 pediatric FUMHD cases and the results of methotrexate treatment have been successful together with systemic steroids in 13 patients [4–16] (Table 1). Cyclosporine and cyclophosphamide were used in addition to the methotrexate treatment in two patients with severe systemic involvement. Herron et al. have reported clinical recovery with the addition of cyclosporine 3 mg/kg/day to methotrexate 15 mg/week in an FUMHD case accompanied by sepsis and ARDS [9]. Rosman et al. reported control of FUMDH with central nervous system vasculitis using systemic steroids and 15 mg/week methotrexate treatment supported by 1000 mg/m^2^ cyclophosphamide treatment. They also emphasized that systemic steroids and methotrexate were added again to control the cutaneous findings that flared at the 5-year follow-up [16]. We observed that methotrexate was given in a dose of 7,5–20 mg/week for FUMDH cases. Neither our case nor any of patients reported in the literature developed liver dysfunction or blood level abnormalities secondary to the methotrexate treatment.

The primary mechanism of action of methotrexate is inhibiting DNA, RNA, thymidylate, and protein synthesis through dihydrofolate reductase inhibition. In addition, it is also effective in lymphoproliferative disorders through its anti-inflammatory features as a result of its effects on T cell activation [17]. FUMDH is considered to be within the lymphoproliferative disorder spectrum and therefore the effect of methotrexate on T cells may be responsible for these results in FUMHD cases.

In conclusion, we think that methotrexate treatment is more effective and reliable treatment option than steroid therapy for pediatric FUMHD cases. Therefore, methotrexate should be considered as first-line treatment in FUMHD.

## Figures and Tables

**Table 1:** Literature review.

Reference (first author)	Age	Sex	Systemic involvement	Therapy	Outcome
Lopez-Estebaranz, 1993 [4]	18 y	M	Liver dysfunction	SS, ATB, MTX, and PUVA	Cure
Fink-Puches, 1994 [5]	16 y	M	(—)	SS, ATB, and MTX	Cure
Romaní, 1998 [6]	12 y	F	(—)	ATB, MTX, and PUVA	Cure
Ito, 2003 [7]	12 y	M	Anemia, abdominal pain, and lymphadenopathy	SS, MTX	Cure
Tsianakas, 2005 [8]	9 y	M	(—)	SS, ATB, and MTX	Cure
Herron, 2005 [9]	8 y	F	Sepsis, DIG, ARDS, and gastrointestinal hemorrhage	SS, MTX, ATB, and cyclosporine	Cure
Pyrpasopoulou, 2007 [10]	17 y	F	Sepsis, anemia, and diarrhea	SS, ATB, IVIG, acyclovir, and MTX	Cure
Helbling, 2009 [11]	17 y	M	Lymphadenopathy	ATB, MTX	
Zhang, 2010 [12]	12 y	M	Pulmonary involvement	SS, ATB, and MTX	Cure
Kaufman, 2012 [13]	21 m	F	Laryngeal edema	SS, MTX	Cure
Kaufman, 2012 [13]	22 m	F	(—)	SS, ATB, acyclovir, and MTX	
Perrin, 2012 [14]	34 m	M	(—)	SS, ATB, acyclovir, dapsone, IVIG, and MTX	Cure
Lin, 2012 [15]	11 y	M	Fever, arthritis, fatigue	SS, MTX	Cure
Rosman, 2013 [16]	11 y	M	Central nervous system vasculitis	SS, ATB, MTX, and cyclophosphamide	Cure
Our patient	11 y	M	Liver dysfunction	SS, ATB, and MTX	Cure

F: female, M: male, y: year, m: month, SS: systemic steroid, ATB: antibiotic, MTX: methotrexate, IVIG: intravenous immunoglobulin, and PUVA: psoralen UVA.
